# Awareness and practices of Arab oncologists towards oncofertility in young women with cancer

**DOI:** 10.3332/ecancer.2022.1388

**Published:** 2022-05-12

**Authors:** Loay Kassem, Nawfel Mellas, Marwan Tolba, Matteo Lambertini, Karima Oualla

**Affiliations:** 1 Clinical Oncology Department, Faculty of medicine, Cairo University, Giza 12555, Egypt; 2 Medical Oncology Department, Hassan II University Hospital, Mohamed Ben Abdellah University, Fes, Morocco; 3 Radiation Oncology Division, McGill University, Montreal, QC, Canada; 4 Department of Medical Oncology, U.O.C Clinica di Oncologia Medica, IRCCS Ospedale Policlinico San Martino, Genoa, Italy; 5 Department of Internal Medicine and Medical Specialties (DiMI), School of Medicine, University of Genova, Genoa, Italy

**Keywords:** oncofertility, female, cancer, breast cancer, fertility preservation

## Abstract

**Background:**

Cancer in young women is a major health problem in the Middle Eastern and North African population. We explored the awareness, barriers and practice of Arab oncologists towards oncofertility.

**Methods:**

Oncologists from Arab countries treating female cancer patients were invited to complete a 30-item web-based questionnaire that explores oncologists’ demographics, available techniques and barriers to oncofertility.

**Results:**

170 oncologists working in 9 different Arab countries responded to the questionnaire. Among the responders, 89 (52.4%) were from Egypt and the central region, 60 (35.3%) were from North Africa and 21 (12.4%) were from the Gulf region.

While most participants considered a dedicated training ‘necessary’, only 43 oncologists (25.3%) received a formal training. Only 17 participants (10%) had a fertility clinic in their centre, 44 (25.9%) and 13 (7.6%) had to refer patients to other centres or other cities, respectively. A total of 96 oncologists (56.5%) did not have access to a fertility preservation service.

Out of 147 responders, 79 (53.7%) offered fertility preservation only in patients presenting with early disease and 38 (25.9%) did not offer fertility preservation. In terms of proposed strategies, 50 responders (29.4%) offered embryo cryopreservation, 79 (46.5%) oocyte cryopreservation and 48 (28.2%) ovarian tissue cryopreservation.

**Conclusion:**

A large gap exists between international clinical practice guidelines and current practices of fertility preservation in Arab countries. Barriers to optimum service delivery include the lack of physician awareness/training, unavailability of some advanced techniques and a lack of dedicated fertility clinics within the cancer centres.

## Introduction

The population distribution in Arab countries is much younger compared to the Western world: more than half (54%) of the population is younger than 25 years, 40% is from 25 to 60 years and only 6% is older than 60 years [1]. This is one of the reasons why the incidence of cancer at a young age is much higher compared to the Western population [2, 3]. This means that a significant proportion of female patients with cancer are diagnosed during their childbearing age. Unfortunately, in this age group, cancer therapies, especially chemotherapeutic agents, may have a negative impact on female fertility [4, 5]. While improvements in cancer treatment have led to increased survival, paying attention to survivorship issues, such as infertility, is of utmost importance [6].

Fertility is one of the key aspects of quality of life for breast cancer patients diagnosed at childbearing age and is considered one of the pillars of good quality care of cancer when diagnosed at a young age [7]. Despite the reduction in childbirths in the past 10–15 years in most of the world, including Arab countries, the number of births is still much higher in the Middle East and North Africa (total births/woman: 3.2 in 2019) compared to the European union (total births/woman: 1.5 in 2019) [8]. These observations highlight the importance of addressing fertility issues among Arab cancer patients [9].

According to international guidelines, fertility preservation strategies should be discussed with all female patients treated for cancer at a young age. Oncologists should explore treatment-induced infertility risks and pay attention to the patient’s wish in having children, following treatment completion, implementing fertility preservation strategies as early as possible after diagnosis [4, 5].

Several barriers are thought to prevent the access of young cancer patients to fertility counselling and fertility preservation strategies, including low awareness about the issues by the treating oncologist [10, 11]. To assess physicians’ awareness and current practice in Arab countries, we conducted a multinational web-based survey regarding fertility preservation in women diagnosed with cancer at a young age.

## Methods

### Selection of participants

Oncologists (medical, clinical, radiation, surgical and gynaecological) from 15 Arab countries were invited to participate. The invited cohort was generated using a list of practicing physicians who registered in three oncology regional conferences. Participants completed a web-based survey to self-evaluate their knowledge, attitude and behaviour regarding oncofertility care in female cancer patients.

### Survey distribution and data collection

A 30-item questionnaire (see Appendix) was prepared by two authors (LK and KO) independently; then, a consensus on the final list of questions was established. The questionnaire was designed to explore the oncologists’ demographics, availability of fertility preservation methods and barriers to fertility counselling (see Appendix). The link to the survey conducted via Google Forms was distributed to 320 oncologists (medical, surgical, radiation, clinical or gynae-oncologists) via email. The response time window was 30 days and a reminder email was sent to non-responders on day 20.

A consent statement was added to be accepted by the participants before proceeding to the questionnaire.

### Statistical analysis

Descriptive analyses were conducted. Chi-square test was used for the correlations between categorical variables. All *p*-values below 0.05 were considered statistically significant. The raw data were exported to Microsoft Excel and the analyses were conducted using IBM SPSS statistics version 20.

## Results

### Characteristics of the respondents

One hundred and seventy oncologists working in nine different Arab countries responded to the survey out of the invited 320 participants (response rate = 53.1%). The participants worked in different Arab regions, with 88 (51.8%) from Egypt, 60 (35.3%) from North Africa and 21 (12.4%) from the Gulf region. Median age was 35 years (range: 25–65 years) and 99 (58.2%) were female. The participants’ demographics are shown in Table 1.

The median duration of oncology experience was 9 years (range: 1–35 years). The majority (78.2%) were medical or clinical oncologists, and 77.7% worked in an academic environment. Interestingly, while almost all the participants (97.6%) considered a dedicated fertility preservation training as “necessary”, only 43 oncologists (25.3%) received a formal training, 11 (25.6%) of them were through a self-sought independent course and not within the oncology training curriculum. Out of 30 participants, 12 (40%) could describe the duration of the fertility course/training they received to be from 1 to 3 hours. Table 2 summarises the experience and training received participating physicians.

### Fertility counseling: Awareness and referral trends

Regarding their current practices, 89 of 170 participants (52.4%) declared that they refer to clinical practice guidelines about fertility preservation in cancer patients, the majority of which referred to US [12] and European [4, 5] based guidelines, and only 1 (0.6%) participant described the use of a local institutional guideline. Most participating oncologists (71.8%) did not have a prepared infertility consent offered to patients about to start their treatment.

Although the majority (82.4%) of oncologists mentioned that they see more than 10 newly diagnosed young female cancer patient per year, only 17 (10%) had a fertility counselling clinic in their centre/hospital, 44 (25.9%) had to refer patients to other centre or even other cities (13 participants, 7.6%) for fertility counselling, while 96 oncologists (56.5%) did not report the presence of a dedicated fertility preservation service. The physician who usually leads the fertility counseling was the treating oncologist (25.5%), a gynaecologist (54.1%) or both (20.4%). Only 30 out of 147 respondents (20.4%) offered fertility preservation to early and metastatic patients, 79 (53.7%) offered it to women presenting with early disease only, while 38 (25.9%) did not offer fertility preservation to any patient. Table 3 describes the fertility preservation process at the participants’ centres. There were no differences in the awareness and attitude towards fertility preservation between male and female physicians and between younger and older physicians.

### Modalities of fertility preservation

Regarding the usually proposed fertility preservation strategy, 79 (46.5%) were able to offer oocyte cryopreservation, while 142 (83.5%) physicians could offer Leutinizing hormone releasing hormone (LHRH) agonist during chemotherapy to their patients. Embryo cryopreservation and ovarian tissue preservation were not widely adopted (29.4% and 28.2% of the participants, respectively) (Table 3).

### Barriers to fertility preservation

Several barriers were explored in our study. First, the fear of delaying the active treatment (e.g., chemotherapy) for advanced stages of cancer was a major barrier. One hundred and thirty-nine physicians (81.8%) believed that – at least sometimes – the patients could get delayed as a result of referral to fertility clinics. This might be the reason why 53.7% of the physicians only refer patients with early disease and 25.9% do not offer the service to all patients. The second barrier is the technical and financial availability. As mentioned before, advanced techniques are not available at most centres. Of note, 140 physicians (82.4%) stated that LHRH agonist was the only modality financially covered by the conventional health insurance in their practice. Finally, a minority of the participating physicians reported facing relevant religious/cultural (11.2%) or legal barriers (15.3%) to providing fertility preservation techniques to young female cancer patients.

## Discussion

We conducted a survey-based study to assess the awareness and practices of Arab oncologists towards oncofertility. Our study demonstrated some striking findings. First, around three-quarters of the participating oncologists did not receive any sort of training for fertility preservation in young cancer patients. Second, only a minority of oncologists in the Arab world were able to offer an integrated fertility preservation services for female cancer patients. LHRH agonist given during chemotherapy for ovarian function preservation was the only available method for the majority (82.4%) of the participating oncologists. More established fertility preservation techniques are rarely available/affordable.

Our study highlights important gaps in a different approach from other previous existing studies. We invited physicians from different disciplines of cancer care and various Arab countries to gain a wider view of their current attitudes in the oncofertility field. In addition, we explored the practices regarding different types of cancers in young women (not only breast cancer, like most previous reports from the region). According to prior studies, several barriers exist in the way adequate oncofertility counselling is managed [13]. The physician’s education/awareness is just one of them. In addition, patient-related factors may also have a major impact. At the time of diagnosis, many patients focus much less on FP compared to cancer prognosis [14]. Finally, the financial and health system barriers are major factors specially in our situation where most Arab population belong to low and low-middle income countries [13].

While most guidelines suggest that oocyte and embryo cryopreservation are the preferred first-line FP options for young female patients with cancer [4, 5, 7, 15], we found a wide gap in discussing/utilising such techniques. This could be explained by both the lack of a dedicated training and the logistic obstacles towards establishing a multidisciplinary FP team.

Several studies have investigated similar gaps worldwide. A survey-based study carried out among 273 oncologists attending the 2016 BCY and the 2017 St Gallen conferences found that 37% of the participants had never refered to existing guidelines regarding FP in breast cancer and that a significant proportion had major misconceptions regarding the issue [10]. A French study by Sallem *et al* [16] assessed the quality of fertility preservation information given by French oncologists to women treated for cancer. The study was carreid out through an online survey on 102 physicians treating different types of cancers. Surprisingly, only 46% of the surveyed physicians routinely discussed the risk of infertility with patients. Moreover, only 22% referred the patients to a fertility specialist before starting their treatments [16].

Regarding the profile of our participants, the majority were young oncologists and 58.2% were female. A Japanese study that included 434 oncologists treating breast cancer patients found that young female oncologists were more likely to refer patients to a fertility specialist/centre [17]. We did not find this association in our survey.

In the Japanese study, oncologists with higher knowledge scores and those who worked in a multidisciplinary environment were more likely to offer FP. This highlights the importance of establishing a dedicated FP training programme as a part of the oncology residency/fellowship training programmes. Similar to our finding, the risk of disease recurrence/progression and fear of treatment delay were major barriers to offer fertility preservation with breast cancer patients [17].

Few studies have assessed the gaps in Arab countries [18, 19]. A survey-based study among 53 oncologists from Lebanon aimed to assess the oncologists’ awareness and knowledge about fertility preservation for cancer patients. Similar to our study, 94% of the surveyed oncologists considered that fertility preservation should be discussed with patients before their cancer treatment. However, oncologists were more aware of male fertility preservation options than female fertility options [19]. A similar study from Saudi Arabia also confirmed the discrepancy in the FP counselling and services offered between female and male cancer patients [18]. Finally, more attention should be paid to FP in Arab women with cancer, taking into consideration the sensitivity of the issue in Arab countries [9].

Our study is, however, limited by not being able to cover all the Arab countries and all the different practice types. Moreover, and due to the survey-based nature of the study, we were not able to assess the needs of healthcare systems in Arab countries in full depth.

## Conclusion

In conclusion, there is a large gap between the international clinical practice guidelines and the current practices regarding fertility preservation in female cancer patients. The barriers to optimum service delivery in Arab countries include inadequate physician awareness/training and the unavailability of some advanced fertility preservation techniques, in addition to some financial constraints. Adding a dedicated FP training to oncology residency and fellowships, encouraging multidisciplinary practice and collaboration and offering financial coverage of FP techniques are essential to advance oncofertility worldwide, including in Arab countries.

## Conflicts of interest

Loay Kassem received honoraria from Roche, Lilly, Novartis, Pfizer and Sandos; all outside the scope of the submitted work.

Marwan Tolba, Nawfel Mellas and Karima Oualla have no conflicts of interest to disclose.

Matteo Lambertini is a consultant for Roche, AstraZeneca, Novartis and Lilly, and received honoraria from Roche, Lilly, Novartis, Pfizer, Sandoz and Takeda; all outside the scope of the submitted work.

## Ethical considerations

All participating physicians provided informed consent (electronically on the platform) before participation. No identifiable patients or physicians’ information was included in the study.

## Funding

The study did not receive any external funding.

## Figures and Tables

**Table 1.** Characteristics of participating physicians.

Parameter		No.	(%)
**Age of the participant (y**ears**)**	Median (range)	35	(25–65)
**Sex**	Male Female	71 99	(41.8) (58.2)
**Oncology experience (y**ears**)**	Median (range)	9	(1-35)
**Country of birth**	Egypt North Africa Gulf Others	88 59 13 10	(51.8) (34.7) (7.6) (5.9)
**Country of** p**ractice**	Egypt North Africa Gulf Others	88 60 21 1	(51.8) (35.3) (12.4) (0.5)
**Specialty**	Clinical oncologist Gynaecologist Medical oncologist Radiation oncologist Surgical oncologist	74 3 59 23 11	(43.5) (1.8) (34.7) (13.5) (6.5)
**Clinical practice environment**	Academic/University Hospital General hospital National Cancer Institute Private hospital/centre	113 25 19 13	(66.5) (14.7) (11.2) (7.6)

**Table 2.** Experience and training of the participating physicians.

Parameter		No.	(%)
**Years of experience**	<5 years 5–10 years 10–15 years >15 years	48 39 48 35	(28.2) (22.9) (28.2) (20.6)
**Number of young cancer patients/year**	0–10 patients 11–50 patients 51–200 patients >200 patients	10 69 61 30	(5.9) (40.6) (35.9) (17.6)
**Received fertility training**	No Yes	127 43	(74.7) (25.3)
**Type of fertility training**	Within the curriculum Self-sought course	32 11	(74.4) (25.6)
**Duration of fertility training**	1–3 hours >3 hours	12 18	(40.0) (60.0)
**Do they see fertility training necessary?**	No Yes	4 166	(2.4) (97.6)

**Table 3.** Fertility preservation process at the participants’ centres.

Parameter		No.	(%)
**Physician initiates discussion**	Always Often Sometimes Never	62 52 52 4	(36.5) (30.6) (30.6) (2.4)
**Patient initiates discussion**	Always Often Sometimes Never	16 35 107 12	(9.4) (20.6) (62.9) (7.1)
**Is an infertility consent available?**	No Yes	122 48	(71.8) (28.2)
**Is fertility preservation part of routine practice?**	No Yes	68 102	(40.0) (60.0)
**Is there a dedicated fertility clinic?**	Yes, in same centre Yes, in nearby centre Yes, in another city No	17 44 13 96	(10.0) (25.9) (7.6) (56.5)
**Fertility preservation available to which patients?**	All, early and metastatic Early only None	30 79 38	(20.4) (53.7) (25.9)
**Can fertility preservation referral cause a delay in starting treatment?**	Always Often Sometimes Never	12 30 97 31	(7.1) (17.6) (57.1) (18.2)
**Available fertility preservation strategies**	Embryo preservation Ovarian tissue preservation Oocyte preservation LHRHa during chemotherapy	50 48 79 142	(29.4) (28.2) (46.5) (83.5)
**Reimbursed fertility preservation strategies**	Embryo preservation Ovarian tissue preservation Oocyte preservation LHRHa during chemotherapy	9 13 19 151	(5.2) (7.6) (11.2) (88.8)

## Appendix: questionnaire

### Fertility preservation strategies for female cancer patients in Arab countries

Thank you for agreeing to participate in this study. By scrolling down, you provide your consent for sharing your non-identifiable responses to this survey.

This is a survey-based study designed to assess the awareness and availability of fertility preservation strategies for female patients treated of different types of cancer. Our target group are oncologists and onco-surgeons who treat young female cancer patients.

* Required

1. Your age *
2. Your Gender *
  *Mark only one oval.*
  - Male
  - Female
3. Country of birth *
4. Country of Practice *
5. What is your specialty? *
  *Mark only one oval.*
  - Medical oncologist Radiation
  - oncologist Surgical oncologist
  - Clinical oncologist (both medical oncologist and radiation oncologist) Gynaecologist
  - Other: ______________________
6. What is your clinical practice environment? *
  *Check all that apply.*
  - National Cancer Institute
  - Academic/University Hospital
  - General Hospital
  - Private Hospital Other:
  - ______________________
7. How long is your oncology experience? *
  (Duration in years)
8. Have you ever had a dedicated training/education on fertility preservation in cancer patients? *
  *Mark only one oval.*
  - Yes
  - No
9. If you had a dedicated training, please specify the type of the training/education you had:
  *Check all that apply.*
  - As part of my oncology training/curriculum
  - A dedicated self sought course.
  - Other: ______________________
10. If you had a dedicated training, How long that training have lasted?
  (Duration in hours)
11. Would you consider this training/education on fertility preservation important and necessary? *
  *Mark only one oval.*
  - Yes
  - No
12. Do you use any clinical practice guideline for fertility preservation in cancer patients? *
  *Mark only one oval.*
  - Yes
  - No
13. If Yes, which guidelines?
14. How many new young female cancer patients (≤ 40 years) do you see in your clinical practice annually? *
  (Number)
15. How often young female patients would initiate discussion by themselves about fertility preservation? *
  *Mark only one oval.*
  - Never
  - Sometimes
  - Often
  - Always
16. Do you have a pre-prepared infertility consent form offered to newly diagnosed young cancer patients? *
  *Mark only one oval.*
  - Yes
  - No
17. How often do you initiate discussion about the possible treatment-related infertility in young cancer patients before starting systemic treatment? *
  *Mark only one oval.*
  - Never
  - Sometimes
  - Often
  - Always
18. Are fertility preserving strategies part of your routine therapy plan for young cancer patients? *
  *Mark only one oval.*
  - Yes
  - No
19. Do you have a dedicated fertility clinics at you practice? *
  *Mark only one oval.*
  - Yes, at the same oncology center
  - Yes, at another nearby center
  - Yes, at another city
  - No
20. If you have fertility preserving consultations at your Institute/hospital, who guides them?
  *Check all that apply.*
  - Gynecologist
  - Oncologist
  - Other: ______________________
21. To which patients fertility preserving procedures are offered at your Institute/hospital?
  *Mark only one oval.*
  - All patients, early & metastatic
  - Only for early cases
  - Not available to any
22. Among newly diagnosed young female cancer patients, what is the percentage who are offered embryo cryopreservation? *
  (%), if not available, please answer with: 0
  ______________________
23. Among newly diagnosed young female cancer patients, what is the percentage who are offered oocyte cryopreservation? *
  (%), if not available, please answer with: 0
  ______________________
24. Among newly diagnosed young female cancer patients, what is the percentage who are offered ovarian tissue cryo-preservation? *
  (%), if not available, please answer with: 0
  ______________________
25. Among newly diagnosed young female cancer patients, what is the percentage who are offered Ovarian suppression with LHRHa during chemotherapy? *
  (%), if not available, please answer with: 0
  ______________________
26. Which of the following fertility preserving methods are financially covered for majority of cancer patients (e.g. state insurance)? *
  “You can choose more than one answer”
  *Check all that apply.*
  - Embryo cryopreservation Oocyte
  - cryopreservation Ovarian tissue
  - cryopreservation
  - Ovarian suppression with LHRHa during chemotherapy
27. How often do you meet any religious or cultural restrictions to fertility preservation strategies in cancer patients? *
  *Mark only one oval.*
  - Never
  - Sometimes
  - Often
  - Always
28. Do you have any legal barrier to fertility preservation strategies in cancer patients?*
  *Mark only one oval.*
  - Yes
  - No
29. Do you have access to a psychological counseling during the fertility preservation process? *
  *Mark only one oval.*
  - Yes
  - No
30. Could referral to fertility counseling clinic/service at your practice cause a delay in starting treatment in poor prognosis (urgent) cases? *
  *Mark only one oval.*
  - Never
  - Sometimes
  - Often
  - Always
